# Beneficial effects of a novel shark-skin collagen dressing for the promotion of seawater immersion wound healing

**DOI:** 10.1186/s40779-017-0143-4

**Published:** 2017-10-27

**Authors:** Xian-Rong Shen, Xiu-Li Chen, Hai-Xia Xie, Ying He, Wei Chen, Qun Luo, Wei-Hong Yuan, Xue Tang, Deng-Yong Hou, Ding-Wen Jiang, Qing-Rong Wang

**Affiliations:** 1 The PLA Key Laboratory of Biological Effect and Medical Protection on Naval Vessel Special Environment, Naval Medical Research Institute, Shanghai, 200433 China; 2 College of Food Science and Technology, Shanghai Ocean University, Shanghai, 201306 China; 3 Research Center of TCM Processing Technology, Zhejiang Chinese Medical University, Hang Zhou, 311401 China

**Keywords:** Anti-seawater immersion dressing, Shark skin collagen, Seawater immersion wound, Wound healing

## Abstract

**Background:**

Wounded personnel who work at sea often encounter a plethora of difficulties. The most important of these difficulties is seawater immersion. Common medical dressings have little effect when the affected area is immersed in seawater, and only rarely dressings have been reported for the treatment of seawater-immersed wounds. The objective of this study is to develop a new dressing which should be suitable to prevent the wound from seawater immersion and to promote the wound healing.

**Methods:**

Shark skin collagen (SSC) was purified via ethanol de-sugaring and de-pigmentation and adjusted for pH. A shark skin collagen sponge (SSCS) was prepared by freeze-drying. SSCS was attached to an anti-seawater immersion polyurethane (PU) film (SSCS + PU) to compose a new dressing. The biochemical properties of SSC and physicochemical properties of SSCS were assessed by standard methods. The effects of SSCS and SSCS + PU on the healing of seawater-immersed wounds were studied using a seawater immersion rat model. For the detection of SSCS effects on seawater-immersed wounds, 12 SD rats, with four wounds created in each rat, were divided into four groups: the 3rd day group, 5th day group, 7th day group and 12th day group. In each group, six wounds were treated with SSCS, three wounds treated with chitosan served as the positive control, and three wounds treated with gauze served as the negative control. For the detection of the SSCS + PU effects on seawater-immersed wounds, 36 SD rats were divided into three groups: the gauze (GZ) + PU group, chitosan (CS) + PU group and SSCS + PU group, with 12 rats in each group, and two wounds in each rat. The wound sizes were measured to calculate the healing rate, and histomorphology and the immunohistochemistry of the CD31 and TGF-β expression levels in the wounded tissues were measured by standard methods.

**Results:**

The results of Ultraviolet-visible (UV-vis) spectrum, Fourier-transform infrared (FTIR) spectrum, circular dichroism (CD) spectra, sodium dodecyl sulfate polyacrylamide gel electrophoresis (SDS-PAGE), and amino acid composition analyses of SSC demonstrated that SSC is type I collagen. SSCS had a homogeneous porous structure of approximately 200 μm, porosity rate of 83.57% ± 2.64%, water vapor transmission ratio (WVTR) of 4500 g/m^2^, tensile strength of 1.79 ± 0.41 N/mm, and elongation at break of 4.52% ± 0.01%. SSCS had significant beneficial effects on seawater-immersed wound healing. On the 3rd day, the healing rates in the GZ negative control, CS positive control and SSCS rats were 13.94% ± 5.50%, 29.40% ± 1.10% and 47.24% ± 8.40%, respectively. SSCS also enhanced TGF-β and CD31 expression in the initial stage of the healing period. The SSCS + PU dressing effectively protected wounds from seawater immersion for at least 4 h, and accelerated re-epithelialization, vascularization and granulation formation of seawater-immersed wounds in the earlier stages of wound healing, and as well as significantly promoted wound healing. The SSCS + PU dressing also enhanced expression of TGF-β and CD31. The effects of SSCS and SSCS + PU were superior to those of both the chitosan and gauze dressings.

**Conclusions:**

SSCS has significant positive effects on the promotion of seawater-immersed wound healing, and a SSCS + PU dressing effectively prevents seawater immersion, and significantly promotes seawater-immersed wound healing.

## Background

Wounded personnel who work at sea often encounter a plethora of difficulties. The most important of these difficulties is seawater immersion. Seawater immersion can exacerbate wounds due to its low temperature, high sodium content, hyperosmolality, and large microorganism population [1]. Rarely dressings have been reported for the treatment of seawater-immersed wounds, but common medical dressings have little effect on the prevention of seawater immersion. A suitable dressing for the treatment of wounded persons who are faced with seawater immersion should not only promote wound healing but also protect the wound from exposure to seawater.

Wound healing consists of several overlapping stages including inflammation, cell proliferation and migration, angiogenesis, re-epithelialization, and reconstruction of the extracellular matrix [2]. Wound dressing materials should be flexible, permeable to gas, and capable of preventing water loss. Many collagen-related products have been developed for wound healing purposes [3, 4]. Type I collagen, a major component of the extracellular matrix, plays an important role in maintaining tissue homeostasis, biological integrity, and structural mechanics through continuous remodeling [5]. Collagen is a good base material for potential dressings, and also has low antigenicity, good biocompatibility, and the ability to promote cell proliferation and attachment. Collagen is also a good chemoattractant for cells required for granulation tissue formation [6]. Collagen and collagen-based matrix materials are the most commonly used biomaterials in skin, connective tissue, and nerve tissue engineering [3]. However, because of Creutzfeldt-Jakob’s disease, foot and mouth disease, and aesthetic and religious issues, the demand for collagen from terrestrial animals has decreased. Alternatively, collagen from fish is considered to be more suitable. Production of fish collagen adds significant value not only to fish processing but also to other pharmacological industries [7]. Blue sharks (*Prionace glauca*) are widely distributed in the Pacific Ocean and East China Sea.

In this study, an acid-soluble shark skin collagen (SSC) was isolated from the skin of blue sharks, and a shark skin collagen sponge (SSCS) was developed. The physicochemical properties of SSCS were subsequently evaluated. The effects of SSCS, a dressing composed of SSCS and an anti-seawater immersion PU film (SSCS + PU) were studied in rat models, and their effects on wound healing, histomorphology and immunohistochemistry of CD31 as well as TGF-β expression in wounded tissues were measured.

## Methods

### Animals

Male SD albino rats were purchased from Sino-British SIPPR/BK Lab. Animal Co. Ltd. The rats were maintained under conditions of standard lighting (12:12 h light–dark cycle), temperature (20–22°C) with freely available food and water. The study was approved by the Ethical Committee of the Naval Medical Research Institute on Animal Care in accordance with the guidelines of the Ministry of Science and Technology of the People’s Republic of China (The Guidance of Experimental Animal Welfare, 2006).

### Extraction of SSC and preparation of SSCS

Skins of blue sharks (*Prionace glauca*) were obtained from Yueqing Marine Organism Health Products Co., Ltd. The frozen shark skin was thawed in running water, and cut into small pieces (1.0 × 1.0 cm^2^). To remove non-collagenous proteins, shark skin pieces were mixed with 0.1 mol/L NaOH at a solid: solvent ratio of 1:10 (*w*/*v*) and continuously stirred for 6 h. Twenty percent ethanol was added at a ratio of 1:10 (w/v) to defat the skin for 24 h, and then, 85% ethanol was added at a ratio of 1:10 (*w*/*v*) to de-sugar and de-pigment the skin for 4 h. The prepared skin pieces were then washed thoroughly with distilled water, and soaked in 0.5 mol/L acetic acid at a ratio of 1:12 (w/v) for 4 h, homogenized, and continuously stirred for 16 h at 4°C. The skin pieces were then centrifuged at 20,000×*g* for 30 min at 4°C. The supernatants were adjusted to pH 7.0, and centrifuged at 20,000×*g* for 30 min at 4°C. The remaining pellet was SSC [8]. The SSC pellet was re-dissolved in 0.5 mol/L acetic acid with a solid:solvent ratio of 1:4 (w/v), and dialyzed against running water for 1 day and distilled water for 2 days until a neutral pH was reached. The gelatinous collagen was transferred to a Petri dish, and freeze-drying. SSCS was subsequently generated.

### Assessment of the biochemical properties of SSC

For Ultraviolet-visible (UV-vis) spectroscopy measurements, SSC was dissolved in 0.5 mol/L acetic acid to a concentration of 1 mg/ml, and then subjected to UV-vis measurement. The spectrum was obtained by scanning at a wavelength range of 190–400 nm at a scan speed of 2 nm/s at room temperature [9].

Fourier-transform infrared (FTIR) spectroscopy measurements from 4000 to 400 cm^−1^ were performed using a FTIR spectrometer (NEXUS470, NICONET, US) at room temperature. The lyophilized SSC was mixed with KBr, thoroughly ground in an agate mortar, and pressed into a 1 mm pellet for FTIR measurement [8, 10].

For circular dichroism (CD) measurement, SSC was dissolved in 0.1 mol/L acetic acid to a concentration of 200 μg/ml and placed in a quartz cell. CD spectra measurements were performed with a CD spectrometer (ASCO J-815, Japan) at various temperatures of 20, 25, 30, 35 and 40°C at wavelengths of 190–280 nm and a scan speed of 2 cm/min. The denaturation temperature (Td) was determined as the temperature at which the change in ellipticity (θ) was half complete [9, 11]. To determine the SSC denaturation temperature, the rotatory angles at fixed wavelengths of 221 nm ([θ]_221_) and 224 nm ([θ]_224_) were measured.

Sodium dodecyl sulfate polyacrylamide gel electrophoresis (SDS-PAGE) of SSC was performed following the Laemmli method [11]. Samples were prepared under reducing and non-reducing conditions through the addition or exclusion of β-mercaptoethanol in the loading buffer. Samples and HiMark™ pre-stained protein standard (Life Technologies, US) were loaded onto an 8% polyacrylamide separating gel and 5% stacking gel.

For amino acid analysis, SSC was hydrolyzed in 6 mol/L HCl at 110°C for 24 h. Amino acid compositions were analyzed using an amino acid analyzer (Hitachi L-8900 Amino Acid Analyzer, Tokyo Japan). The amino acid contents were expressed as the number of residues/1000 residues [7, 10].

### Assessment of the physicochemical properties of SSCS

The morphology of SSCS was studied by scanning electron microscopy (SEM, Phenom Prox, Holland). A sponge of 1 mm^2^ was examined under an accelerating voltage of 20 kV [9].

The porosity rate of SSCS was evaluated using the trimmed samples of 5 cm^2^× 0.5 cm in ethanol [12], and calculated according to the following formula:

$$ \mathrm{Porosityrate}\;\left(\%\right)=\mathrm{Vc}/\mathrm{Vm}\times 100=\left[\left({\mathrm{W}}_{24}-{\mathrm{W}}_0\right)/\uprho \right]/\mathrm{Vm}\times 100 $$

Where Vm is the total volume of SSCS (cm^3^), Vc is the pore volume (cm^3^), W_24_ is the weight (g) of SSCS after incubation with ethanol for 24 h, W_0_ is the original weight (g) of SSCS, and ρ is the density of ethanol (0.79 g/ cm^3^).

The swelling behavior of SSCS in different solutions was analyzed. Samples of SSCS approximately 1 cm^2^ in size were incubated in distilled water, a 0.9% NaCl solution, PBS, and a solution similar to wound exudate according to EN 13726–1 [13] at room temperature to the maximum swelling ratio. After incubation, the excess surface liquid was removed, and the samples were weighed (W_i_). The weight of the initial dry sample was noted as W_0_. The swelling ratio of SSCS (*n* = 5) was calculated using the following equation:

$$ \mathrm{Swellingratio}=\left({\mathrm{W}}_{\mathrm{i}}-{\mathrm{W}}_0\right)/{\mathrm{W}}_0 $$

Where the water vapor transmission ratio (WVTR) of SSCS was determined according to Gorczyca et al. [13] by monitoring the mass of evaporated water from SSCS and by measuring the weight loss from a water-filled homemade permeability cup. The permeability cups were filled with 20 g of deionized water, and test samples were fixed on the opening of the cup. The permeability cups were weighed and placed in a desiccator at 37°C, which equilibrated the desiccator to a relative humidity of approximately 20%. The WVTR values were calculated using the following equation:

$$ \mathrm{WVTR}=\mathrm{m}/\left(\mathrm{A}\times \varDelta \mathrm{t}\right) $$

Where m is the weight (g) of the lost water at the specified time period, Δt is the time period (h), and A is the effective transfer area (m^2^). Each of the measurement was performed in triplicate.

The tensile strength of SSCS was characterized as previously described [14]. Three rectangle-shaped specimens of 10 mm × 50 mm were prepared. The tensile strength (MPa) and percentage of elongation at break (%) were measured using as electronic fabric strength tester (YG-B-026G-500, Wenzhou Darong Textile Instrument Co., Ltd., China).

### Detection of the promotional effects of SSCS on seawater-immersed wound healing

Twelve male SD albino rats, weighing 250 ± 20 g, were randomly divided into four groups: the 3rd day group, 5th day group, 7th day group and 12th day group, with three rats in each group. After preparing the skin on the back of the rats, four round wounds (diameter 0.6 cm) were created in each rat with a hole puncher to the depth of the loose subcutaneous tissue, and the wounded rats were immersed in seawater at 32°C for 4 h. Then, two of the four wounds in each rat were covered with SSCS, one wound was covered with chitosan (CS) dressing as the positive control, and another wound was covered with gauze (GZ) dressing as the negative control. All of the dressings were sterilized with 25 kGy ^60^Co γ-ray irradiation. On the 3rd, 5th, 7th, and 12th days, the wound areas were imaged by photography and analyzed using Image J software (NIH, US). The healing rate of each wound was calculated using the following equation:

$$ \mathrm{Healingrate}\;\left(\%\right)=\left[\left({\mathrm{A}}_1-{\mathrm{A}}_{\mathrm{t}}\right)/{\mathrm{A}}_1\right]\times 100 $$

Where A_t_ and A_1_ are the wound areas on the detected day and wounded day (first day), respectively.

For histological analysis, the harvested wound tissue samples were fixed in 4% formaldehyde solution at 4°C, dehydrated with a graded series of ethanol solutions, embedded in paraffin, and sequentially sectioned at 4 μm thickness. The wound tissue sections were stained with hematoxylin and eosin (HE) to analyze of re-epithelialization and granulation tissue formation. Furthermore, wound tissue sections from three rats of each group at each time point were immunostained to detect the expressions levels of CD31 and TGF-β [15–17]. After blocking with blocking buffer, the sections were incubated with diluted CD31 or TGF-β primary antibody in a wet chamber overnight at 4°C. After washing the slides with PBS, the slides were incubated with secondary antibodies for 1 h at room temperature. After washing with PBS 5 times, the slides were mounted with immunostaining mounting media and covered with cover slips. Fluorescent images were captured using a Leica CH-9435 fluorescent microscope, and the expressions levels of CD31 or TGF-β were semi-quantitatively analyzed by Image J.

### Assessment of the effect of the SSCS + PU dressing on protection from seawater immersion and wound healing

SSCS was attached to an anti-seawater immersion PU film (developed by Naiqier Biotechnology Co. Ltd. China) to compose the anti-seawater immersion dressing (SSCS + PU), and GZ and CS were attached to the PU film as a negative control (GZ + PU) and a positive control (CS + PU), respectively. All of the dressings were sterilized with 25 kGy of ^60^Co γ-ray irradiation. Thirty-six male SD albino rats weighing 250 ± 20 g were randomly divided into three groups: the GZ + PU group, CS + PU group and SSCS + PU group, with 12 rats in each group. After depilation and sterilization of the back of rats, one round wound (diameter 0.8 cm) was created in each rat with the hole puncher. The wounds of each group were covered with the corresponding dressings and immersed in seawater at 32°C for 4 h. On the 3th, 5th, 8th, 11th, and 13th days, the wound sizes were imaged by photography and analyzed using Image J software. The healing rate of the wound was calculated. Three rats per group were selected and euthanized on the 5th, 8th, 11th, and 13th days, and the wound skin tissues were removed for histomorphology analysis and immunohistochemistry of the CD31 and TGF-β levels as described in Assessment of the physicochemical properties of SSCS section.

### Statistical analysis

Statistical analysis was performed by one-way analysis of variance (AVOVA) followed by LSD *t*-test using SPSS (version 17.0). All of the values were expressed as the mean ± SD. *P* < 0.05 were considered to be statistically significant.

## Results

### Biochemical properties of SSC

**Fig. 1:**
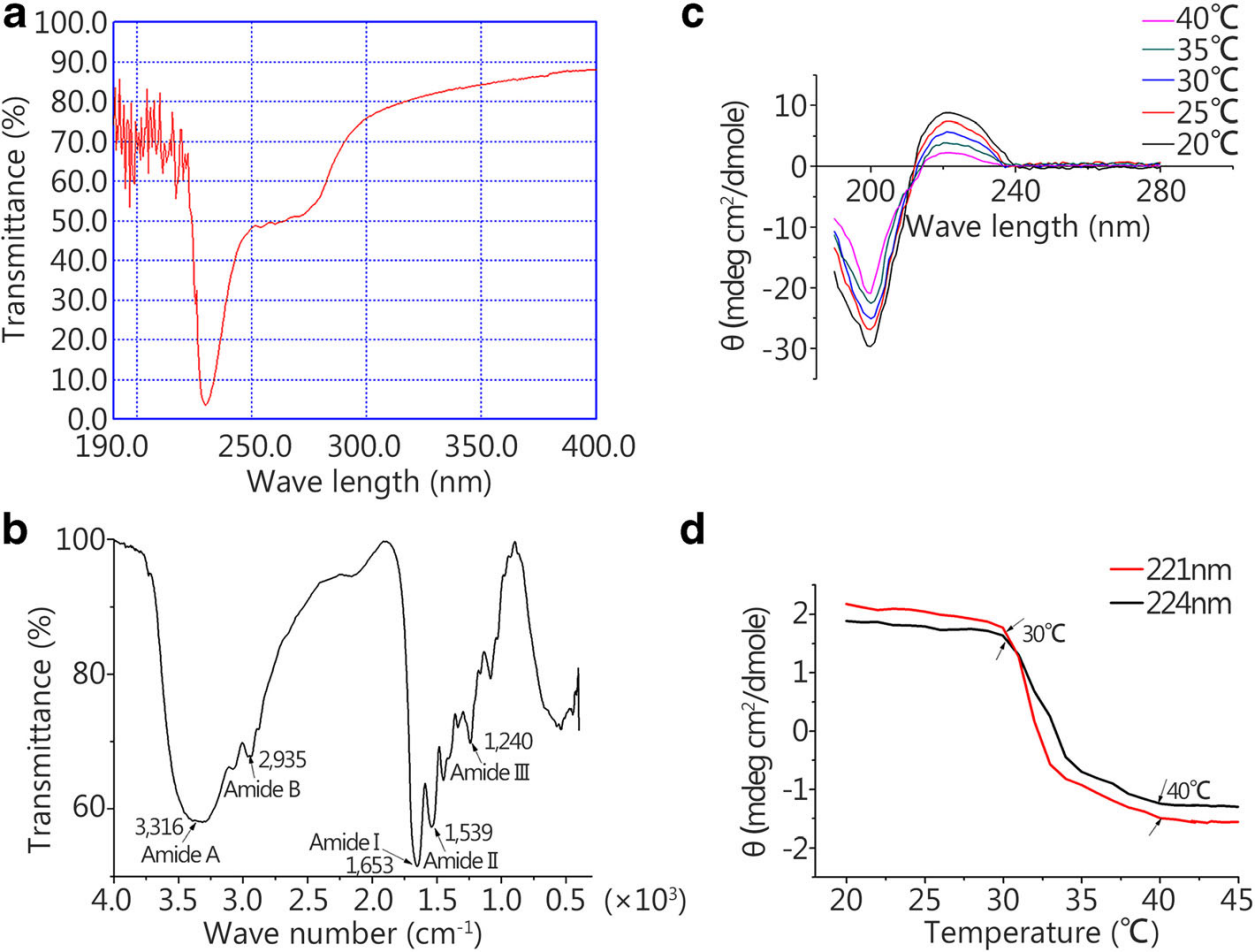
Physicochemical properties of SSC. **a** UV absorption spectrum of SSC. **b** The Fourier-transform infrared spectrum of SSC. **c** CD spectra of SSC at 20, 25, 30, 35 and 40°C. **d** Effect of temperature on CD spectra of SSC at 221 nm and 224 nm

The results of spectrum analyses of SSC are shown in Fig. 1. UV-vis spectrum analysis showed a high intensity absorbance at 230 nm and no high absorption peak at 280 nm (Fig. 1a), which suggested that highly pure collagen had been produced.

The FTIR spectrum of SSC is shown in Fig. 1b. The amide A band was found at 3316 cm^−1^, and it was generally associated with the N-H stretching vibration, showing the existence of hydrogen bonds. The amide B band was found at 2935 cm^−1^, representing asymmetrical stretching of CH_2_. The sharp amide I band was observed at 1653 cm^−1^ and was associated with the C = O stretching vibration or a hydrogen bond coupled with COO^−^. The amide I region was mainly used for the analysis of the secondary protein structure. The characteristic peak of the amide II region was observed at 1539 cm^−1^. The amide II vibration modes were attributed to the N-H in-plane bend and C-N stretching vibration. The FTIR spectrum indicated the triple helix structure of SSC.

CD spectra of SSC from 20 to 40°C are shown in Fig. 1c. It was shown that the rotatory maxima were at 221 nm and the minima were at 200 nm, and a consistent crossover point (zero rotation) was found at approximately 210 nm, which is characteristics of a triple helical protein conformation. The spectra in Fig. 1d show the corresponding mean molar ellipticity change from 20 to 45°C at 221 and 224 nm. The [θ]_221_ and [θ]_224_ values both decreased with the increase in temperature due to the denaturation of the triple helical structure. The denaturation temperature (Td) was 35°C.

**Fig. 2:**
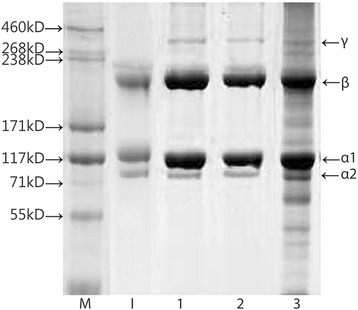
SDS-PAGE analysis of SSC. M: HiMark protein standard; I. type I collagen from calf skin. Lanes 1 and 2: SSC under non-reducing and reducing conditions. 3: SSC from desalinization condition under reducing conditions. α1, α2, β and γ were the α1 chain, α2 chain, β chain and γ chain of shark skin collagen, respectively

SDS-PAGE of SSC was performed out under reducing and non-reducing conditions and is displayed in Fig. 2. SSC contained as α_1_-chain, α_2_-chain, β-chain and γ-chain, and their molecular weights were approximately 117, 101, 200, and 340 kD, respectively. The electrophoretic bands of SSC in the presence and absence of β-mercaptoethanol were similar, which indicated that three were no disulfide bonds in the collagen. Additionally, it was observed that the collagen from the desalinization process had several heterogeneous bands, which suggested that purer collagen could be obtained by adjusting the pH than by removing salt.

The amino acid composition of SSC and compared with collagen of brown banded bamboo shark skin [8] and tilapia skin [18] is shown in Table 1. SSC contained 33% glycine, and the other primary amino acids in SSC were alanine, proline, hydroxyproline and glutamic acid. While the levels of tyrosine and histidine were very low and cysteine was not detected, the levels of proline and hydroxyproline were important to the structural integrity of collagen, and the amount of imino acid (proline and hydroxyproline) in SSC was 18.1%. SSC had low methionine and insignificant cysteine levels. These results suggested that SSC was type I collagen.

**Table 1:** Amino acid composition of SSC and compared with collagen of brown banded bamboo shark skin [8] and tilapia skin [18] (residues/1000 amino acid residues)

Amino acid	Collagen of blue shark skin	Collagen of brown banded bamboo shark skin	Collagen of Tilapia skin
Aspartic acid	42	42	41
Threonine	24	23	24
Serine	41	41	33
Glutamic acid	76	77	68
Glycine	330	318	319
Alanine	118	105	118
Valine	25	25	16
Cysteine	0	1	0
Methionine	11	12	6
Isoleucine	21	18	8
Leucine	26	24	2
Tyrosine	3	3	2
Phenylalanine	14	14	27
Lysine	26	29	24
Histidine	8	7	5
Arginine	54	51	52
Hydroxyproline	76	93	77
Proline	105	111	113
Imino acid (Pro + Hyp)	181	204	190

### Physicochemical characteristics of SSCS

**Fig. 3:**
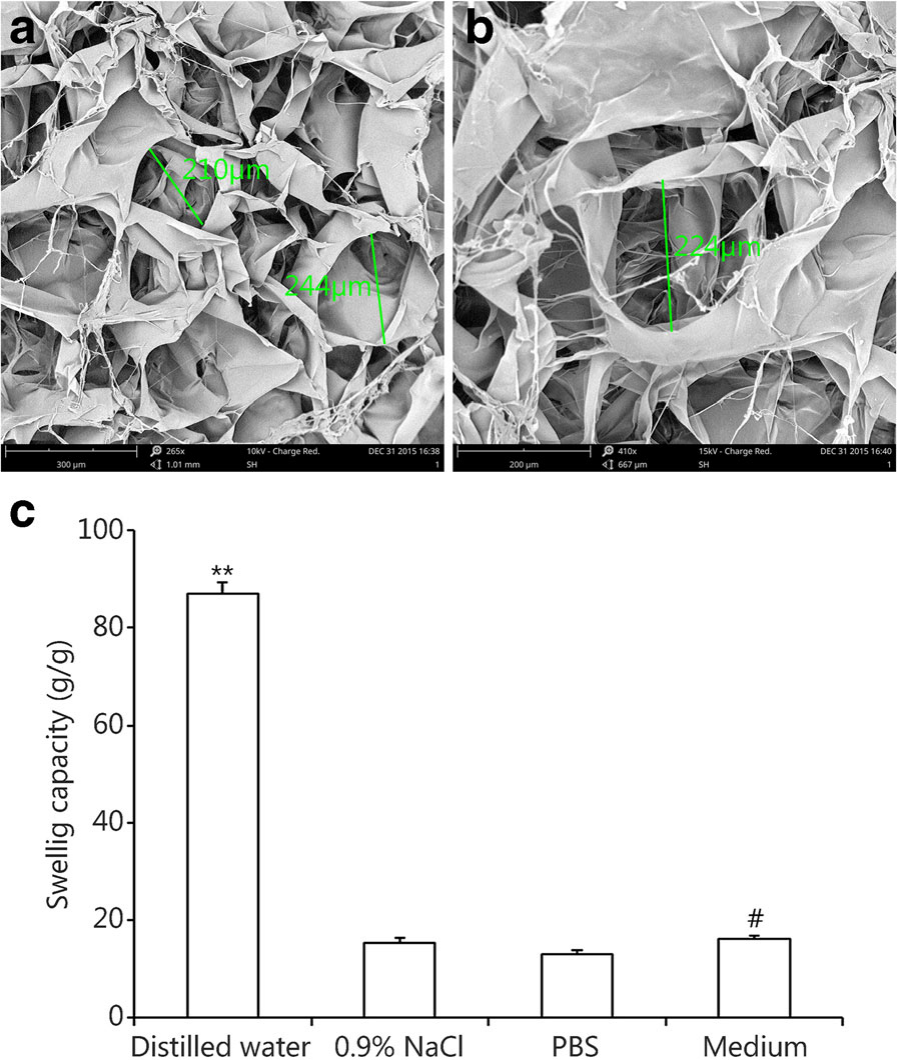
Characteristics of SSCS. **a** and **b** SEM images of SSCS (**a**: 265×, **b**: 410× magnification). **c** Swelling capacity of SSCS in water, 0.9% NaCl, PBS and a medium with a salt composition similar to wound exudate. *P < 0.01 distilled water compared with the other three solutions, #*P* < 0.01 compared with PBS

SEM images of SSCS are shown in Fig. 3a and b. Lyophilized SSCS had homogeneous porous structure with sizes of approximately 200 μm, and the porosity rate was 83.57% ± 2.64%. The high porosity of SSCS resulted in good air permeability and wound exudate absorption. Generally, water absorption and retention properties are critical to a wound dressing because the dressing must absorb large amounts of wound exudate and prevent bacterial invasion. The water absorption capacity of SSCS was evaluated by immersion in different solutions, and the results are shown in Fig. 3c. The swelling ratios were 86.96 ± 2.30 g/g in distilled water, 15.4 ± 0.96 g/g in 0.9% NaCl, 13.13 ± 0.75 g/g in PBS and 16.18 ± 0.70 g/g in a medium with a salt composition similar to wound exudate. The swelling ratio in distilled water was much higher than in the other three solutions (*P* < 0.01), and the swelling ratio in the medium solution was higher than in PBS (*P* < 0.05). The WVTR of SSCS was approximately 4500 g/m^2^, which did not affect the normal rate of water loss from the wound. An ideal wound dressing should maintain suitable mechanical properties. The tensile strength of SSCS was 1.79 ± 0.41 N/mm, and the elongation at break was 4.52% ± 0.01%, which are critical to maintaining the integrity of the wound dressing.

### SSCS promoted the healing of seawater-immersed wounds in rats

#### The healing rate of seawater-immersed wounds

**Fig. 4:**
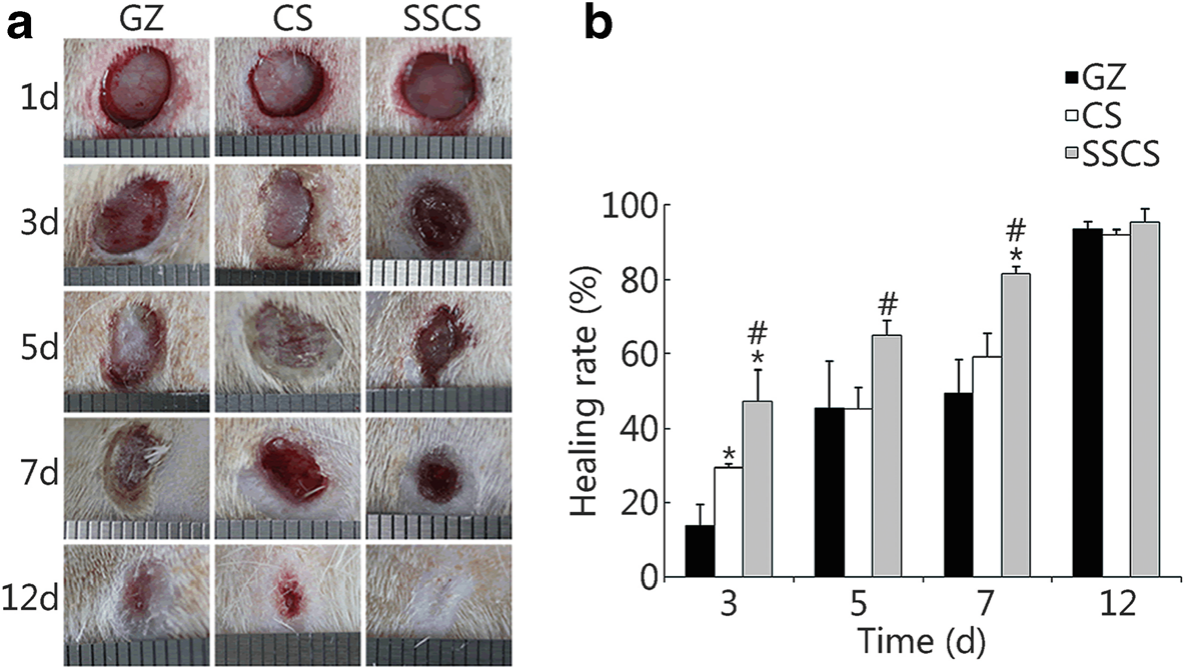
The promotion effects of SSCS on the healing rate of seawater-immersed wounds in rats. **a** A schematic diagram of wound healing. **b** Wound healing rate on the 3rd, 5th, 7th and 12th days. *P < 0.05 compared with the GZ group; #*P* < 0.05 compared with the CS group

The effects of SSCS on the healing of seawater-immersed wounds in rats are shown in Fig. 4. The wound healing progression (Fig. 4a) and wound healing rate (Fig. 4b) showed that the wound area decreased at each time point in all three groups. On the 3rd day, the wound healing rate in the GZ negative control, CS positive control and SSCS were 13.94% ± 5.50%, 29.40% ± 1.10% and 47.24% ± 8.40%, respectively, and the wound healing rate of the SSCS group was significantly higher than those of the CS group and GZ group (*P* < 0.05). On the 5th day, the healing rates in the GZ group, CS group and SSCS group were 45.22% ± 12.80%, 45.08% ± 5.85%, and 64.96% ± 3.90%, respectively. On the 7th day, the healing rates in the GZ group, CS group and SSCS group were 49.31% ± 9.10%, 59.25% ± 6.20% and 81.71% ± 1.70%, respectively, and the healing rate of the SSCS group was higher than those of the CS group and GZ group (*P* < 0.05). On the 12th day, the wounds of the SSCS group were almost completely healed, the surface of the healing wound was smooth, and the wound appeared to be vastly improved compared to the two control groups. These results suggested that SSCS can significantly enhance wound healing progression, and that its promotional effect is superior to the currently clinically used biomaterial dressing made of chitosan and the common gauze dressing.

#### Re-epithelialization and granulation tissue formation of seawater-immersed wounds

**Fig. 5:**
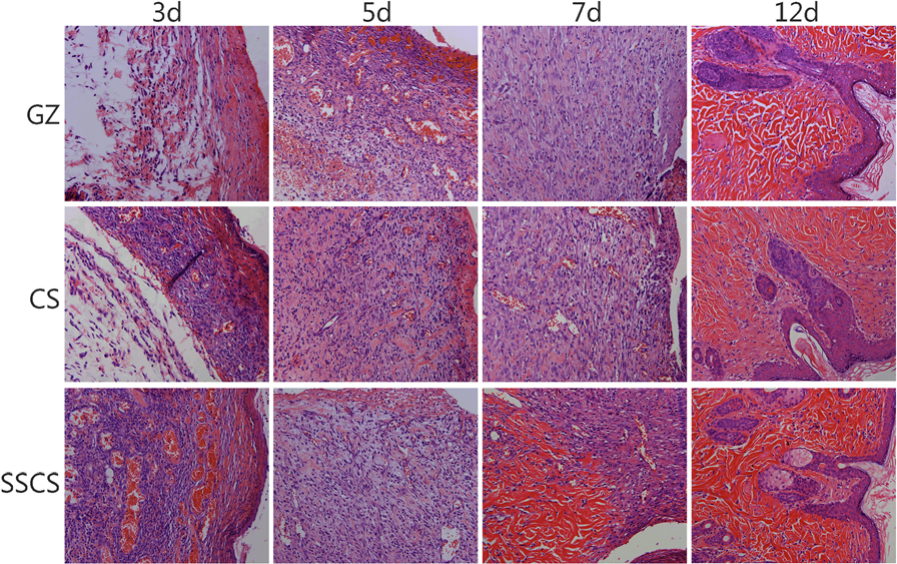
Hematoxylin and eosin stained sections of the seawater-immersed wound regions treated with GZ, CS and SSCS (LSA × 200)

To confirm re-epithelialization and granulation tissue formation in the wound healing progression of seawater-immersed wounds treated by SSCS, skin tissue sections from the wound were stained with HE, and the results are shown in Fig. 5. On the 3rd day, inflammation was observed in all three groups, and the wound regions in the SSCS group had significantly more blood capillaries and akaryocytes than those of the CS positive control group and GZ negative control group. On the 5th day, there was obvious granulation tissue growth and partial fibroblasts in the SSCS group and CS group, but the GZ group showed a small amount of fibroblasts and multiple inflammatory infiltrate at the edge of the wounds. On the 7th day, the new epidermis was formed to a greater extent in the SSCS group and CS group. The new muscle tissue grew visibly in the SSCS group, but it was not observed in the other two control groups. On the 12th day, all of the groups demonstrated granulation tissue formation and dermal remodeling. The wound surfaces treated by SSCS were much smoother and the new muscle tissue was more full-grown.

#### CD31 expression in seawater-immersed wound tissue

**Fig. 6:**
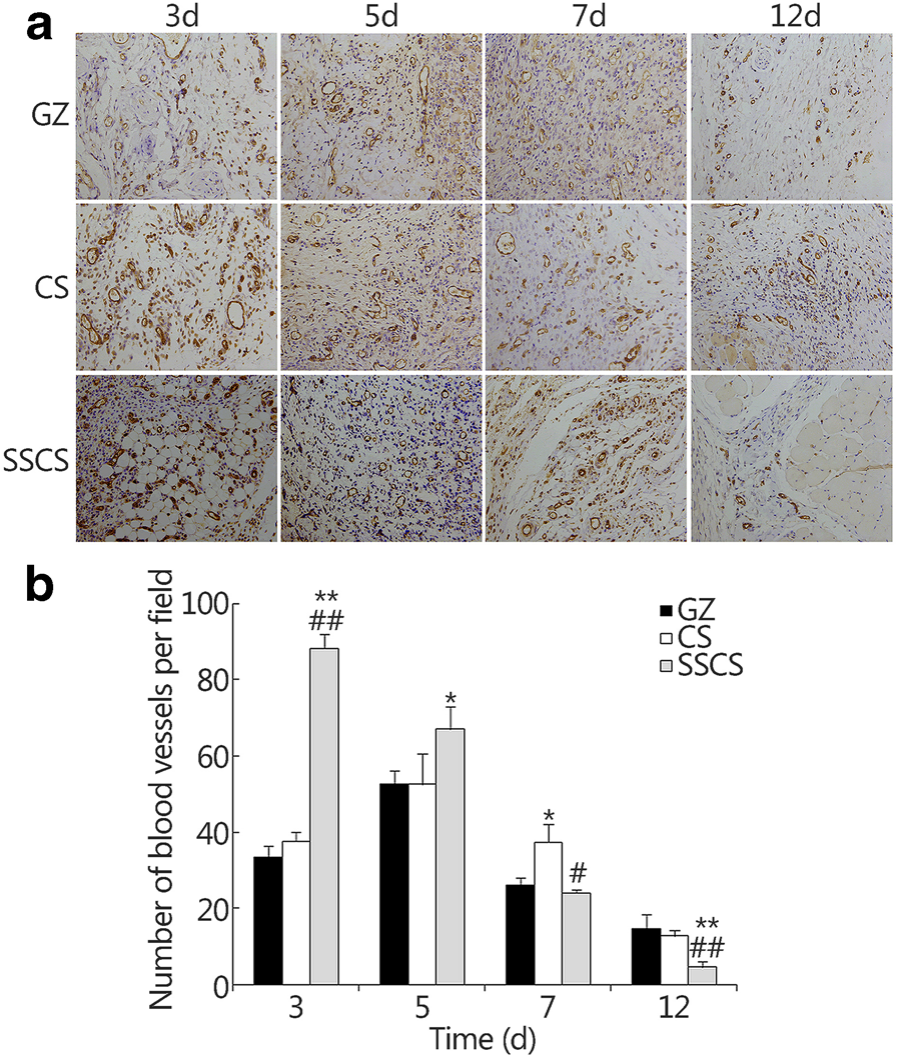
The promotional effects of SSCS on the expression of CD31 within seawater-immersed wound tissues. **a** Analysis of immunopositivity for CD31 in the seawater-immersed wound of the experimental groups on the 3rd, 5th, 7th and 12th days (LSA × 200). **b** Quantitative analysis of the new blood vessels as revealed by CD31 immunohistostaining within the wound on the 3rd, 5th, 7th and 12th days. *P < 0.05, **P < 0.01 compared with the GZ group; #*P* < 0.05, ##*P* < 0.01 compared with the CS group

Angiogenesis is essential to wound healing. Expression of CD31 in endothelial cells can be used to indicate the level of angiogenesis in the wound tissues. In the initial stage, wound healing proceeds faster if vascularization is initiated earlier. However, in mid-anaphase of wound healing, excess vascularization is not conducive to fibroblast growth and re-epithelialization of the wound tissue. The effect of SSCS on CD31 expression is shown in Fig. 6. On the 3rd day, the CD31 expression levels in the GZ group, CS group, and SSCS group were 33.33 ± 3.05, 37.67 ± 2.52, and 88.00 ± 3.61, respectively. On the 5th day, the expression levels in the same three groups were 52.67 ± 3.51, 52.67 ± 8.14 and 67.00 ± 6.00, respectively, and on the 7th day, they were 26.00 ± 2.00, 37.33 ± 4.73 and 24.00 ± 1.00, respectively. On the 3rd day and the 5th day, CD31 expression levels in the SSCS group were significantly higher than those in the negative and the positive control groups (*P* < 0.01, respectively). On the 12th day, there was markedly less blood vessel content in the SSCS group, but there was still some CD31 expression in the CS group and GZ group. This result indicates that the SSCS group had almost completed wound healing by the 12th day with less new blood vessel growth, but the CS group and GZ group, whose wound healing processes were still ongoing, showed more angiogenesis. These results suggested that SSCS could have significant promotional effects on angiogenesis, as revealed by the increased expression of CD31 during the initial stages of wound healing.

#### TGF-β expression in seawater-immersed wound tissue

**Fig. 7:**
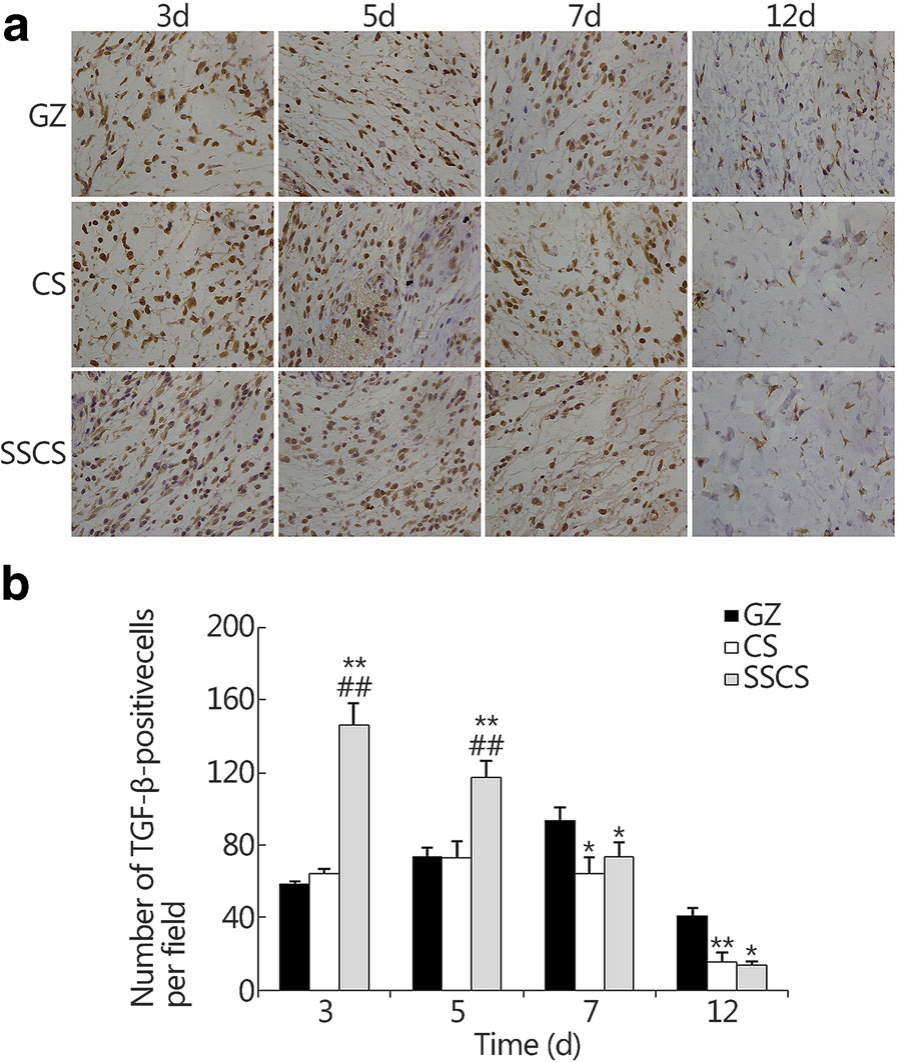
The promotional effects of SSCS on the expression of TGF-β. **a** Analysis of immunopositivity for TGF-β in the seawater-immersed wounds on the 3rd, 5th, 7th and 12th days (LSA × 400). **b** Quantitative analysis of the immunohistostained TGF-β within the wound. *P < 0.05, **P < 0.01 compared with the GZ group; ##*P* < 0.01 compared with the CS group

The effect of SSCS on the expression of TGF-β in seawater-immersed wound tissue is shown in Fig. 7, and the TGF-β expression levels were identified by brown staining. On the 3rd day of the wound healing period, the TGF-β expression levels in the GZ group, CS group, and SSCS group were 58.30 ± 1.53, 64.00 ± 3.00 and 146.33 ± 11.67, respectively. On the 5th day, the expression levels of the same three groups were 73.67 ± 5.13, 72.67 ± 9.50 and 117.30 ± 9.07. On the 7th day, the expression levels were 93.67 ± 7.23, 64.00 ± 9.54 and 73.33 ± 8.08, respectively. On the 12th day, there was reduced TGF-β expression, but the level in the GZ group was still 41.00 ± 4.36 which was significantly higher than in the SSCS group and CS groups (*P* < 0.01, respectively). The TGF-β expression levels in the SSCS group were much higher than those in the GZ group and CS group on the 3rd and 5th days (*P* < 0.01). Moreover, the expression level in the SSCS group reached its highest value on the 3rd day, whereas it reached its highest level on the 5th day in the CS group and on the 7th day in the GZ group. These results suggest that SSCS should have much higher promotional effects on TGF-β expression in the initial stage of wound healing, and should decrease its expression in the final stage of wound healing, which partially explains why SSCS can stimulate wound healing and decrease scar formation.

### The SSCS + PU dressing protected wounds from seawater immersion and promoted wound healing

#### Anti-seawater immersion and wound healing rate

**Fig. 8:**
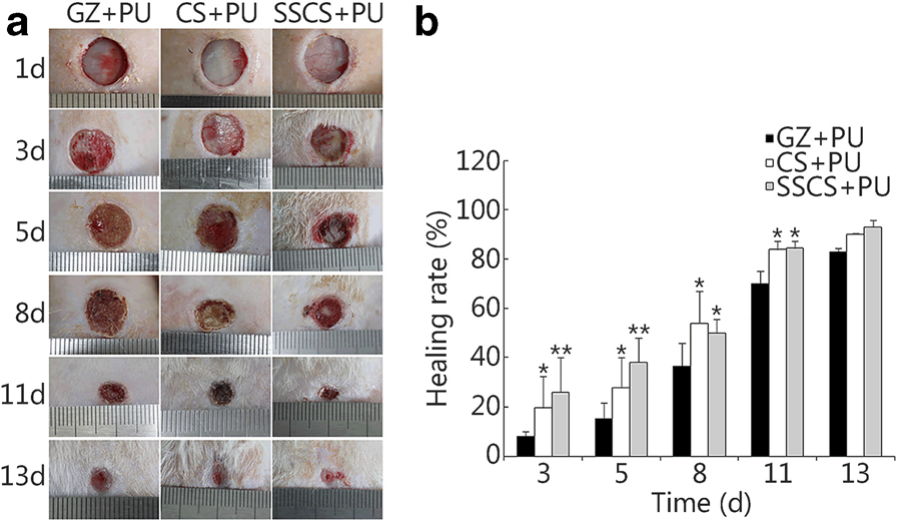
The promotional effects of the SSCS + PU dressing on the healing rate of anti-seawater-immersed wounds in rats. **a** Schematic diagram of wound healing. **b** Wound healing rates on the 3rd, 5th, 8th, 11th and 13th days. *P < 0.05, **P < 0.01 compared with the GZ + PU group

Seawater immersion may intensify wound damage. Conventional medical dressings have little effect on anti-seawater immersion. A dressing that can not only promote wound healing, but also efficiently protect wounds from seawater immersion is desperately needed. An SSCS + PU dressing was prepared from SSCS and an anti-seawater immersion PU film, and its effects on preventing seawater immersion and promoting wound healing are shown in Fig. 8. After immersion in seawater for 4 h, no seawater was observed to permeate the covered wound area, which means that the constructed PU film can effectively protect the wound from seawater immersion, and no side effects were observed in the animals. Early during the healing period, the SSCS + PU dressing markedly stimulated wound healing. On the 3rd, 5th, 8th, and 11th days, the wound healing rates of the SSCS + PU groups were 25.90% ± 14.02%, 38.02% ± 9.98%, 49.97% ± 5.63%, and 84.48% ± 2.73%, respectively. The healing rates of the CS + PU groups were 19.74% ± 2.57%, 27.73% ± 12.26%, 53.94% ± 12.97%, and 83.86% ± 3.50%, respectively, but the healing rates of the GZ + PU groups were 8.17% ± 2.02%, 15.17% ± 6.48%, 36.32% ± 9.42% and 69.87% ± 5.28%, respectively. The wound healing rates of the SSCS + PU groups were significantly higher than those of the other two groups, and more granulation and newly formed epidermis tissues were clearly observed at the margin of the wound in the SSCS + PU groups on the 3rd and 5th days. On the 13th day, the healing rates of the three treated groups had no significant differences (*P* > 0.05). These results suggest that the application of the SSCS + PU dressing not only effectively protect the wound from seawater immersion, but also have more significant promotional effects on wound healing.

#### Re-epithelialization and granulation tissue formation

**Fig. 9:**
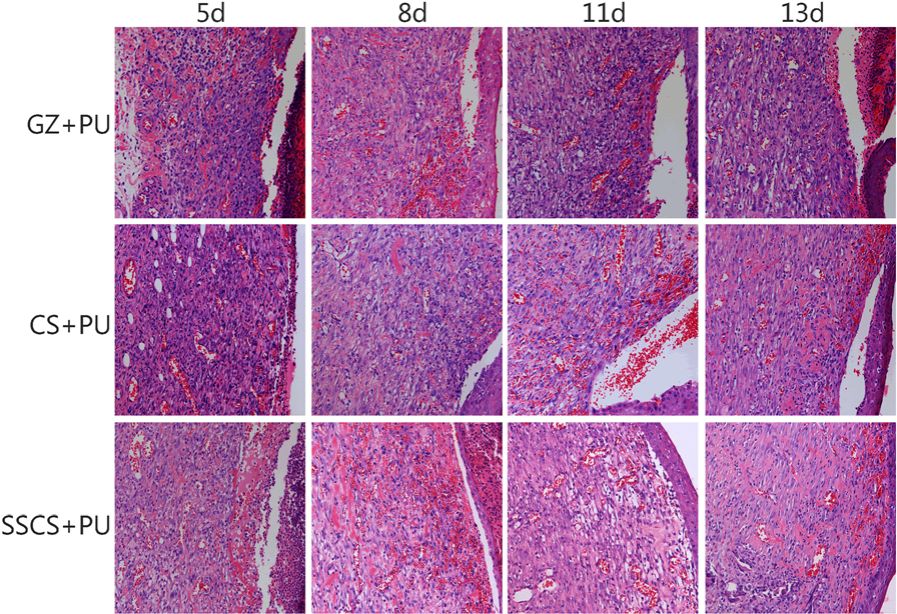
Hematoxylin and eosin stained sections of SSCS + PU anti-seawater-immersed dressing. (LSA × 200)

Histological examination demonstrated the general morphology of the skin layers during the process of wound healing. The results are shown in Fig. 9. On the 5th day, there were several new blood capillaries and akaryocytes in the SSCS + PU and CS + PU groups, and strong inflammatory reaction occurred in the GZ + PU group. On the 8th day, there were more fibroblasts and fewer inflammatory cells in the SSCS + PU group than the GZ + PU group and CS + PU group. On the 8th and the 11th days, the new epidermis was well formed and more new granulation tissue was observed in the SSCS + PU group than in the other two groups. On the 13th day, all of the groups demonstrated new epidermis formation and dermal remodeling. The new epidermis was well integrated with the dermis in SSCS + PU group.

#### CD31 expression

**Fig. 10:**
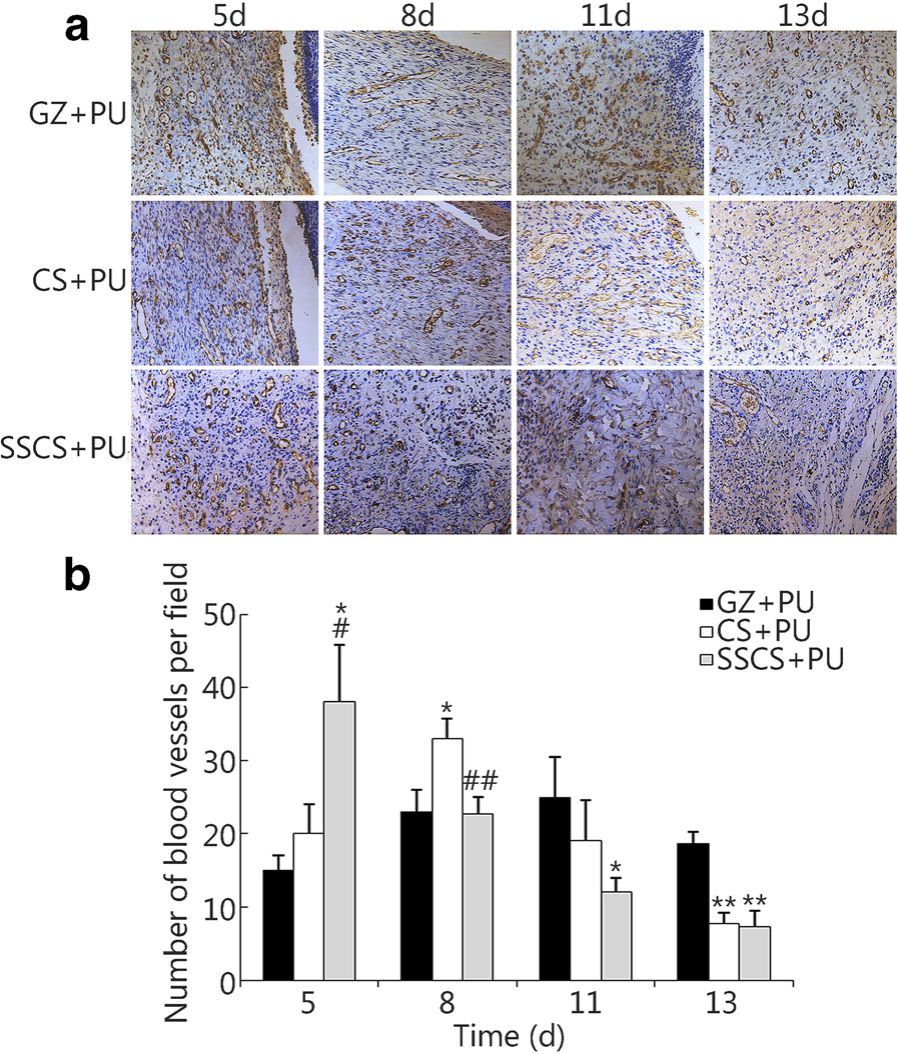
The promotional effect of SSCS + PU dressing on the expression of CD31 within anti-seawater-immersed wound tissues. **a** The analysis of immunopositivity for CD31 in the blood vessels within the anti-seawater-immersed wounds of the experimental groups on the 5th, 8th, 11th and 13th days (LSA × 200). **b** Quantitative analysis of the immunohistostained blood vessel levels within the wound tissues. *P < 0.05, **P < 0.01 compared with the GZ + PU group; #*P* < 0.05, ##*P* < 0.01 compared with CS + PU group

The results of CD31 expression levels are shown in Fig. 10. On the 5th day, the CD31 expression levels in the GZ + PU group, CS + PU group and SSCS + PU group were 15.00 ± 2.00, 20.33 ± 4.04 and 38.00 ± 7.81, respectively, and the SSCS + PU group levels were significantly higher than those of the CS + PU group and GZ + PU group (*P* < 0.05, respectively). On the 8th day, CD31 expression levels in the GZ + PU group, CS + PU group and SSCS + PU group were 23.00 ± 3.00, 33.00 ± 2.65 and 22.67 ± 2.31, respectively. There was a clear reduction in the SSCS + PU group (*P* < 0.01 compared with that on the 5th day). On the 11th day, the blood vessel levels in the GZ + PU group, CS + PU group and SSCS + PU group were 25.00 ± 5.57, 19.00 ± 5.57 and 12.00 ± 2.00, respectively. CD31 expression level in the SSCS + PU group reached its highest level on the 5th day of the wound healing period, where the highest levels in the CS + PU group and GZ + PU group were on the 8th day and the 11th day, respectively. On the 13th day, CD31 expression levels in the SSCS + PU group and CS + PU group were decreased to their lowest levels, but that in the GZ + PU group was still higher than the other two groups (*P* < 0.01, respectively). These results showed that the SSCS + PU dressing stimulates expression of CD31, and the highest peak value was observed much earlier. These suggest that the SSCS + PU dressing should promote the angiogenesis in the early stages of wound healing and should decrease it in the later stages, and the dressing was also found to promote wound healing and reduce the formation of scars.

#### TGF-β expression

**Fig. 11:**
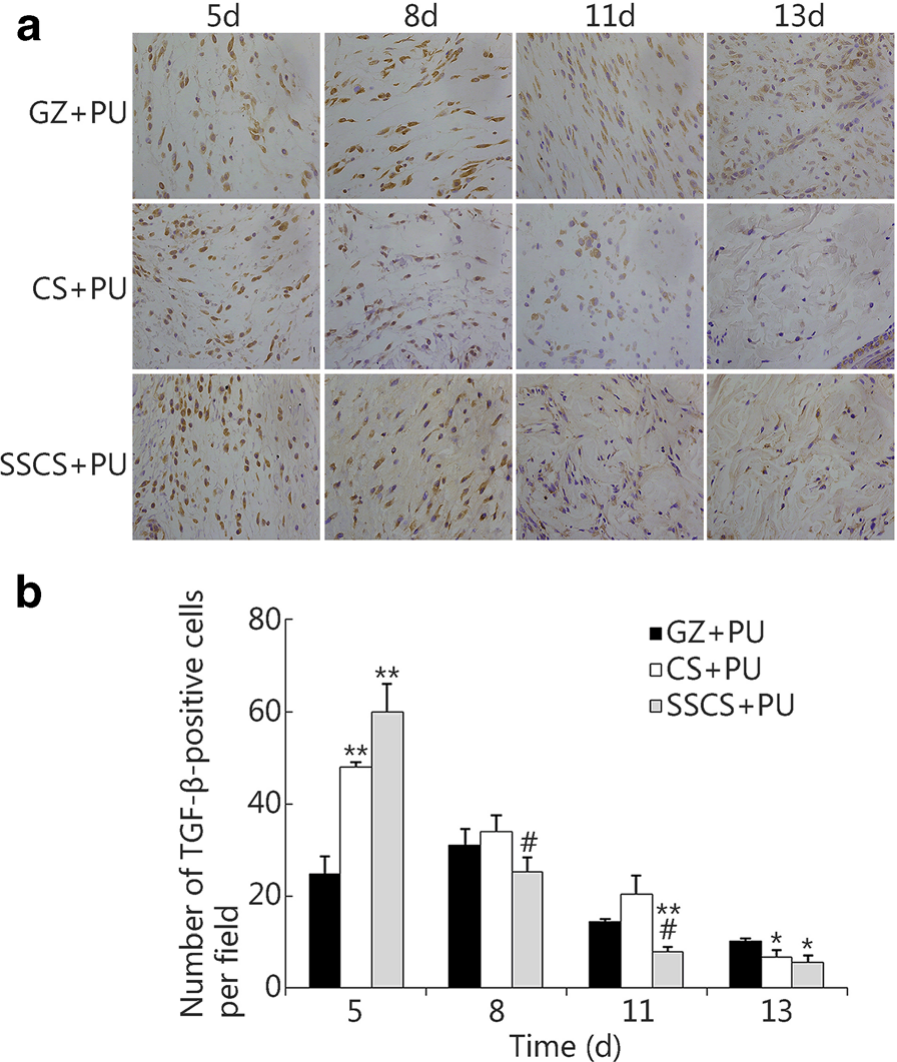
The promotional effect of the SSCS + PU dressing on the expression of TGF-β. **a** The analysis of immunopositivity for TGF-β within the anti-seawater-immersed wounds of the experimental groups on the 5th, 8th, 11th and 13th day (LSA × 400). **b** Quantitative analysis of the immunohistostained TGF-β levels within the wound. *P < 0.05, **P < 0.01 compared with the GZ + PU group; # *P* < 0.05 compared with the CS + PU group

The results of TGF-β expression analysis are shown in Fig. 11. On the 5th day, the TGF-β expression levels in the SSCS + PU group and CS + PU group reached their highest levels of 60.00 ± 6.24 and 48.00 ± 1.00, respectively, and were significantly higher than that in the GZ + PU group (24.67 ± 3.79, *P* < 0.01). On the 8th day, TGF-β expression in the GZ + PU group reached its highest level of 31.00 ± 3.61, and the expression levels in the SSCS + PU group and CS + PU group significantly decreased. On the 11th day, TGF-β expression was lower in all three groups, and the SSCS + PU group showed much lower expression level than the CS + PU group (*P* < 0.05) and GZ + PU group (*P* < 0.01). On the 13th day, TGF-β expression in the GZ + PU group was 10.00 ± 0.58, and was significantly higher than in the other two groups (*P* < 0.05). These results suggested that the SSCS + PU dressing should promote the expression of TGF-β at an earlier time point and to a greater extent.

## Discussion

Wounds immersed in seawater are more complicated and severe than common wounds encountered terrestrially [1]. Studies have shown that wounds can become more edematous and deeper after immersion in seawater. Additionally, vascularization of the wound is delayed, as is the wound healing [19].

TGF-β contributes to wound healing in part through stimulation of vascularization, fibroblast proliferation, myofibroblast differentiation, collagen synthesis, granulation tissue formation, and re-epithelialization [20, 21]. TGF-β can stimulate the proliferation of fibroblasts, and promote the conversion of cellula intersitialis to fibroblasts, and promote the conversion of fibroblasts to myofibroblasts [22]. TGF-β also contract the wound [23]. TGF-β can activate the proliferation of vascular endothelial cells to promote remodeling of the vascellum and emerging granulation tissue in the initial stages of wound healing [24]. However, prolonged expression of TGF-β in the later stage of the healing process can lead to scarring [25]. Philips proposed that the use of TGF-β might accelerate the healing of many types of wounds at specific stages of wound healing [26]. Blood vessels composed of endothelial cells deliver oxygen and nutrients to the cells and accelerate the migration of the requisite cells and humoral factors into the wound site. These processes facilitate the synthesis of collagen and the formation of granulation tissue, as well as facilitate wound healing [27]. TGF-β mediates these processes through two signaling pathways, the SMAD pathway and MAPK/ERK pathway [28]. MAPK/ERK signaling pathway plays an important role in cell proliferation, metabolism and apoptosis [29]. Initiation of Src/ERK signaling by TGF-β is important for the promotion of vascularization in the wound site [30]. SSCS showed significant effects on the expressions levels of TGF-β and CD31 in wounded tissues, especially in the early stages of wound healing, and had remarkable effects on the healing of seawater-immersed wounds, its promotional effect was superior to that of chitosan and gauze. These results suggest that SSCS can advance the wound healing period by at least for2 days compared with the chitosan dressing. The dressing consisting of SSCS attached to an antiseawater-immersed PU film successfully protected wounds from seawater immersion for at least 4 h. This new dressing effectively increased the healing rate, re-epithelialization, and dermal reconstitution of the wound and protected the wound from seawater immersion. The mechanism underlying these effects may, in part, involve increasing expression of TGF-β and promoting angiogenesis, which remains to be studied further in future studies.

Wound healing involving tissue regeneration is a complicated biological process. An effective wound dressing should protect the wound from secondary infection, maintain a moist environment following the absorption of wound exudates, provide adequate gaseous exchange, and exhibit good biocompatibility with tissues and blood [31]. Collagen is a good humectant, and the highest utilization of collagen has been in pharmaceutical applications, including the production of wound dressings [32]. SSC extracted from blue sharks is a type I collagen, and has triple-helical structure. The three-helix structure of collagen is important in medical dressings, which require the stability of collagen dressing and is also helpful for the growth and migration of fibroblasts. It has been reported that a scaffold for skin regeneration should have a pore architecture with a mean pore size between 100 and 200 μm [33]. SSCS had a homogeneous porous structure with a mean pore size of approximately 200 μm and porosity rate of 83.57% ±2.64%, which indicate that SSCS has a good air permeability and wound exudate absorption, and that SSCS is able to maintain a moist environment for wound healing.

## Conclusion

The collagen extracted from blue shark skin is a type I collagen. The novel constructed SSCS + PU dressing has significant effects on wound healing promotion and anti-seawater immersion, and can promote expression of TGF-beta and CD31 in tissues in the early stages of wound healing. This new dressing with wound healing promotion and anti-seawater immersion should be convenient for treating wounds acquired at sea, especially for wounded soldiers serving on warships.

## Abbreviations

CD: Circular dichroism
CS: Chitosan
FTIR: Fourier transform infrared
GZ: Gauze
HE: Hematoxylin and eosin
PU: Polyurethane film
SDS-PAGE: Sodium dodecyl sulfate polyacrylamide gel electrophoresis
SEM: Scanning electron microscopy
SSC: Shark skin collagen
SSCS: Shark skin collagen sponge
UV-vis: Ultraviolet-visible
WVTR: Water vapor transmission ratio
